# Wireless Middleware Solutions for Smart Water Metering

**DOI:** 10.3390/s19081853

**Published:** 2019-04-18

**Authors:** Stefano Alvisi, Francesco Casellato, Marco Franchini, Marco Govoni, Chiara Luciani, Filippo Poltronieri, Giulio Riberto, Cesare Stefanelli, Mauro Tortonesi

**Affiliations:** 1 Department of Engineering, University of Ferrara, 44122 Ferrara, Italy; stefano.alvisi@unife.it (S.A.); francesco.casellato@unife.it (F.C.); marco.franchini@unife.it (M.F.); marco.govoni@unife.it (M.G.); chiara.luciani@unife.it (C.L.); giulio.riberto@unife.it (G.R.); cesare.stefanelli@unife.it (C.S.); 2 Department of Mathematics and Computer Science, University of Ferrara, 44121 Ferrara, Italy; mauro.tortonesi@unife.it

**Keywords:** Internet-of-Things, smart metering, water consumption

## Abstract

While smart metering applications have initially focused on energy and gas utility markets, water consumption has recently become the subject of increasing attention. Unfortunately, despite the large number of solutions available on the market, the lack of an open and widely accepted communication standard means that vendors typically propose proprietary data collection solutions whose adoption causes non-trivial problems to water utility companies in term of costs, vendor lock-in, and lack of control on the data collection infrastructure. There is the need for open and interoperable smart water metering solutions, capable of collecting data from the wide range of water meters on the market. This paper reports our experience in the development and field testing of a highly interoperable smart water metering solution, which we designed in collaboration with several water utility companies and which we deployed in Gorino Ferrarese, Italy, in collaboration with CADF (Consorzio Acque Delta Ferrarese), the water utility serving the city. At the core of our solution is SWaMM (Smart Water Metering Middleware), an interoperable wireless IoT middleware based on the Edge computing paradigm, which proved extremely effective in interfacing with several types of smart water meters operating with different protocols.

## 1. Introduction

The efficient use of natural resources is increasingly important, especially with the current trends towards a larger population mainly concentrated in urban environments (smart cities and megacities) [1,2]. Several impact analyses have proven that smart metering solutions are highly beneficial to society, as they are very effective both in engaging citizens to reduce excessive consumption and in detecting resource waste [3,4,5].

There is a growing interest in smart metering applications [6], that have initially focused on the energy [7] and gas [8] utility markets, which deal with the more expensive resources. Recently, a growing interest has focused on smart metering of water consumption [5]. Initial smart water metering solutions have proved very useful in order to identify and prevent water leakages, which is an increasingly important concern. In fact, while water represents a cheaper commodity than gas in many countries and its leakage does not present any hazard, water leakages in the residential and distribution network are very frequent and difficult to detect. The growing interest in sustainability in modern society has been stimulating and fostering the development of the smart water metering market to fight water leakages [9,10,11].

However, so far, research has dedicated a limited effort to the investigation of smart water metering solutions. This is unfortunate, because smart water metering presents interesting challenges both at the scientific and at the engineering levels. More specifically, we can identify three main problems to address.

First, lacking an open and widely accepted communication standard in the smart water metering market, most companies manufacturing smart water meters propose proprietary solutions. In addition, the smart metering market is quickly evolving from the communications perspective: while a few years ago smart meters only used the Wireless M-Bus communication protocol, more recent meter also adopt LoRa, and the first NarrowBand IoT (NB-IoT) solutions are emerging. This results in vendor and protocol lock-in issues for water utilities, which might lead to high expenditures (both CAPEX and OPEX) and ultimately hinder innovation.

Second, a significant part of the water meters installed at the end user level are of the traditional mechanical type, designed to display water consumption visually and read by a human operator. These “dumb” meters have a very large installation base and still represent a cheap and well tested solution. As a result, they are likely to play a non-negligible role in the water utility market for (at least) the near future. This means that smart water metering solutions designed for large scale deployment need to have the capability to operate with dumb meters, possibly implementing sophisticated automated reading solutions.

Finally, there is the need to address practical aspects connected to real-life deployment of large scale smart water metering solutions. More specifically, how to address radio frequency propagation issues and how to set the reading frequency (a trade off between observability and energy consumption) represent issues of major importance. Reports on practical knowledge developed on the field, which are unfortunately, very rare at the moment of this writing, would help designers to build improved smart water metering solutions.

This paper attempts to bridge this gap by introducing a comprehensive smart water metering solution, which we designed in collaboration with several Italian water utility companies to address specifically the peculiarities and requirements of the smart water utility market. This paper also reports on our extensive field testing experiences. Our solution leverages the Edge Computing approach to distribute data pre-processing functions in proximity of the water meters and takes advantage of modern Commercial Off The Shelf (COTS) IoT technologies, both at the hardware and at the (open source) software level, to reduce costs and facilitate interoperability.

At the core of our solution is SWaMM (Smart Water Metering Middleware), an interoperable wireless IoT middleware that is capable of interfacing with a wide range of smart water meters operating with different protocols. SWaMM Edge Gateway devices running the SWaMM Edge component collect water consumption data from the smart meters, with a dynamically tunable frequency that allows tradeoffs between observability and energy saving, preprocess the data, transcoding it if needed and aggregating it to consolidate transmissions, and forward it to the SWaMM Cloud platform, which further analyzes the data to return useful consumption pattern information both to the users and to the water utility.

Our solution was thoroughly tested in the context of two field experiments. First, we deployed SWaMM to perform the smart water metering of all residential houses in Gorino Ferrarese, Italy. In collaboration with CADF (Consorzio Acque Delta Ferrarese), the water utility serving the city, all the mechanical water meters in Gorino Ferrarese were replaced with smart ones. In addition, we deployed several gateways to retrieve consumption data from the smart water meters and forward it to the SWaMM Cloud platform.

In addition, we tested the capabilities of our solution to integrate with an existing water metering infrastructure by deploying SWaMM to implement the smart water metering of selected residential homes in Ferrara, Italy. More specifically, we selected several residences in Ferrara that represented frequent use cases (apartment in the historical city center, apartment and a single home in the suburbs) with different water meters.

From the initial experimentation to the deployment in the field, SWaMM has shown a remarkable interoperability and was capable of interfacing with a wide range of water meters. Not only SWaMM interfaced with smart meters adopting either the Wireless M-Bus or the LoRa communication protocols but also with traditional mechanical meters retrofitted with an Optical Reader Kit that we designed to integrate SWaMM with older water metering installations. Finally, the analysis of the data collected implemented by the SWaMM platform has proven very effective in detecting leakages.

The rest of the paper is organized as follows. Section 2 describes the smart water metering field, discussing proprietary and open solutions to address water metering data collection, also including a description of different types of water meters available on the market. Section 3 presents the design of our comprehensive and interoperable solution for smart water metering based on the SWaMM wireless IoT middleware. Section 4 discusses the architecture of SWaMM and describes (in detail) the SWaMM Edge Gateway and the SWaMM Optical Reader Kit components, that respectively deal with the data collection and elaboration at the edge and with the retrofitting of dumb water meters. Section 5 provides a thorough evaluation of our solution by reporting the results of several field experiments, including a relatively large deployment in Gorino Ferrarese, Italy, for the monitoring of water consumption in domestic residences. Section 6 discusses the advantages of open and interoperable smart water metering solutions such as SWaMM and their potential impact in a growing and competitive market. Section 7 illustrates related work in the smart metering field, highlighting the differences between those solutions and SWaMM. Finally, Section 8 concludes the paper and discusses future work.

## 2. Smart Water Metering: From Proprietary to Open Solutions

Both providers and consumers present common interests in the smart water metering market [3]. On the one hand, providers are interested in having a fully automated data collection to reduce operational costs associated to human interventions, to produce billing information based on effective/accurate data, and to have a larger control over distribution networks and delivery points. On the other hand, consumers have the opportunity to have frequent water metering data to increase consumption awareness and adapt their behaviour accordingly [4], i.e., avoiding waste of resources and being notified about possible warnings and leakages.

With respect to the energy and gas markets, smart water metering presents important criticalities. In fact, water usually tends to be much cheaper than other commodities (gas, electricity), and thus, resulting in less investments and efforts in the market. Another interesting criticality is that the water meters are usually located in underground and/or protected areas (inspection pits), which do not have an electricity coverage for obvious safety reasons. This results in systems that need to be battery powered and extremely energy efficient to provide at least an acceptable average lifetime period. In addition, these underground areas may also present radio frequency propagation issues, which significantly reduces the strength and the range of wireless transmissions. However, the increasing reduction of water availability [12] due to several factors such as climate changes and population growth [13] and significant technological advances in embedded systems fostered important innovations in smart metering solutions for the water utility market.

### 2.1. Smart Water Meters

Despite smart water metering being a relatively recent practice, there are several smart water meters available on the market. The first generation of smart water meters adopted low power short-range wireless protocols, such as Wireless M-Bus, that operates over the unlicensed spectrum (169 or 868 MHz in Europe). These meters were designed to operate in Remote Meter Reading (RMR) systems, in which data collection can be performed without a dedicated networking infrastructure: operators equipped with portable receivers collect data in the proximity of the smart meters either in walk-by or drive-by mode. RMR systems eliminate the need for physical access or visual inspection of smart meters but do not allow either real-time or automated consumption monitoring.

More recently, a second generation of smart water meters that leverage on low-power long-range wireless protocols, such as LoRa (Long Range), which is designed for a wider communication range both in urban and extra-urban environments, hit the market [14,15]. These meters were specifically designed for Automatic Meter Reading (AMR) systems, in which data collection is fully automated: smart water meters periodically transmit their consumption information to gateway devices that gather the data from the in-range smart meters and retransmit it to the utility management typically using mobile (3G/LTE/4G) communications. To guarantee user privacy, smart water meters encrypt consumption information before the transmission.

Unfortunately, despite the large number of solutions available on the market, the lack of an open and widely accepted communication standard pushed vendors to propose proprietary data collection solutions. The adoption of proprietary solutions presents water utility companies with several significant problems in term of costs, vendor lock-in, and lack of control on the data collection infrastructure.

In fact, vendors typically propose proprietary and often very expensive gateways, that need to be acquired in addition to smart meters and whose cost might account for a non negligible portion of capital expenditure for water utility companies that want to implement an AMR system. This is further exacerbated in case short-range water meters are employed. In fact, while nothing prevents short-range smart meters to be adopted within AMR systems, their limited communication radius would require the deployment of a large number of gateways. Thorough planning and field testing would be needed in order to minimize the number of gateways required to collect data from all the smart meters, and in some cases the deployment of ad hoc signal repeaters to extend the radio coverage of smart meters would be required.

In addition, due to the lack of an open standard, smart meter vendors have typically adopted proprietary protocol specifications to transmit metering data over the network. As new smart meters need to be compatible with the existing monitoring solution, this may easily result in vendor and communication protocol lock-in problems. Lock-in risks are particularly high at the moment of this writing, in which smart water metering could arguably be classified at the “early adopters” stage of the technology life cycle. In fact, solutions on the market are rapidly evolving with respect to lower energy consumption and longer communication range, and the first NarrowBand IoT (NB-IoT) proposals are emerging. As a result, choosing proprietary solutions offered by a vendor now might hinder or preclude the adoption of significantly more convenient water meters from a different vendor in a few years.

Finally, some manufacturers have developed smart meters that are able to communicate both through an open OMS (Open Meter Standard) (https://oms-group.org/en/download4all/oms-specification/) radio protocol and a proprietary protocol, which usually can provide some additional information. Furthermore, on the metering market it is common for manufacturers to provide proprietary platforms designed to receive data from different types of smart meters using proprietary radio protocol (for their own smart meters) and also to collect data using the OMS protocol, and thus, enabling data collection from smart meters that support open standard. A representative example of a similar solution is the one provided by Sensus, which makes use of the proprietary radio protocol SensusRF to collect metering data from Sensus and other types of smart meters communicating via the OMS protocol and operate on frequencies on the 868 MHz band. However, such solution, equipped only with 868 MHz antennae, cannot receive messages from smart meters that operates on different frequencies, such as the WaterTech meters that communicates on frequencies on the 169 MHz band.

The proprietary nature of most smart water metering solutions on the market also has an impact in terms of a limited amount of control for adopters. The lack of access to the software running on gateway devices in particular represents a major obstacle to the implementation of innovative features for customers. For instance, smart metering systems are typically configured to operate with a specified data collection frequency, configured either at manufacturing or at installation time. The possibility to control the behavior of gateways could, for instance, allow to dynamically tune the frequency of data acquisitions, raising it in case a leakage is suspected.

### 2.2. Traditional “Dumb” Water Meters

For many decades, water meter manufactures have used only basic physical principles of measurements in the design of water meters. For instance, the Italian legislation forced, until a few years ago, water utilities and meter manufactures to adopt and develop metering instrumentation based exclusively on mechanical/dynamic measurement principles. This regulations and the low manufacturing costs of this type of water meters reflected in water metering parks mainly composed of traditional “dumb” measuring instruments. Most of the time, this instrumentation is composed of a turbine and mechanical gears. The water passing through the meter makes move the turbine, which is connected to mechanical gears that transform the rotational speed of the turbine in a flow measurement. This measure is then expressed in cubic meters (m^3^), or fractions of m^3^, on the consumption wheel of the water meter’s dial. Therefore, this information must be read on the water meter’s dial and it refers to the total volume (cumulative consumption) of water transited since the installation of the water meter.

Specifically, there are different models of mechanical water meters that differ in terms of the mechanism the meter uses and the typology of account it was designed for (residential or industrial). For example, single-jet meters are designed for residential accounts, multiple jet meters are used both on residential and industrial accounts, and Woltmann meters are used mainly for industrial accounts.

Since the smartness of a metering instrumentation is a characteristic associated to the ability of transmitting metering data and not to the instrumentation itself, it becomes possible to enable “smartness” even on traditional mechanical meters. In fact, in recent years, water metering manufactures have introduced communication modules capable of reading data from a pulse emitter connected to the mechanical meter and transmitting over a wireless radio protocol, e.g., Wireless M-Bus. For example, Sensus produces a mechanical water meter capable of integrating a pulse emitter (https://sensus.com/products/hri-mei/), which is wire connected to a battery-powered Wireless M-Bus radio module (https://sensus.com/products/sensus-pulse-rf/).

However, not every traditional dumb water meter can be equipped with a pulse emitter. In these cases, to enable an automated data collection from the whole metering park, water utilities can adopt two different strategies. The first one consists in massive water meter replacement campaigns, but such solution requires huge investments and operational costs. Finally, the other possible strategy is to equip traditional dumb water meters with an optical reading system that can analyze an image of a water meter’s dial and transform it into a computer-processable consumption value.

The two strategies present different costs. Not considering the costs of adopting a proprietary data collection infrastructure, the cost of a modern smart water meter equipped with a radio module is about EUR 100. Instead, we calculated that retrofitting a traditional mechanical dumb water meter with an optical reader kit requires approximately EUR 30. However, the adoption of one strategy instead of the other mainly depends on management and business decisions of water utilities.

### 2.3. IoT as a Foundation for Open Smart Water Metering Solutions

These considerations call for innovative and highly interoperable solutions, based on open standards and technologies instead of proprietary ones and capable of reading consumption data from a remarkably heterogeneous set of water meters, either of the smart and of the dumb type, and to enable utility companies to adopt different technologies at the same time. For instance, a similar solution would allow companies to adopt new smart meters or communication protocols in a part of their distribution network while the rest of the network would continue to operate with previous technologies.

Fortunately, the astounding progresses achieved by the Internet-of-Things (IoT) revolution have provided inexpensive and high quality tools that represent a terrific foundation upon which to build a comprehensive and interoperable smart water metering solution capable of operating with different types of meters using different open wireless protocols and data formats.

With this regards, wireless IoT communication solutions represent compelling key enabling technologies for smart metering applications. In addition to the already mentioned Wireless M-Bus and LoRa protocols, which are being increasingly adopted by smart water metering manufacturers, there are a range of wireless IoT protocols that might be relevant for smart metering applications, with different capabilities in term of allowed bitrate, range of operation (distance), number of supported devices, and power consumption [16].

Within those, IEEE 802.15.4, and its full stack proprietary extension ZigBee, is widely adopted in low power networks and provides transmissions capabilities over a limited range (10–50 m) and it supports different network topologies: star, peer-to-peer, and cluster [17]. Bluetooth LE is a relatively recent and non backwards compatible version of Bluetooth specification, featuring increased communication range (up to 100 m) and lower power consumption, albeit at the expense of a lower maximum bitrate (1 Mbps) [18]. Another interesting wireless IoT protocol is Sigfox, a proprietary communication technology developed by the eponymous Sigfox company. Unlike LoRa, which is also proprietary but infrastructureless and free to use, Sigfox adopters need to leverage on a communication infrastructure provided by Sigfox, and pay corresponding licensing fees. On the top of some of these protocols it is possible to build larger wireless network infrastructures. For instance, LoRaWAN (Low Power WAN Protocol for Internet-of-Things) provides WAN communications on top of LoRa, which end-devices can use to communicate with gateways [19].

With the only notable exception of Sigfox, all these solutions allow to perform information exchange at the edge of the network on the unlicensed spectrum, and consequently to realize wireless smart metering solutions without depending on the support of network providers/operators or paying licensing fees [20].

In addition, modern Commercial Off-The-Shelf (COTS) hardware solutions are inexpensive and battle tested, ranging from powerful Single Board Computers (SBC) based on ARM microprocessors, which can run full operating systems such as Linux, to very low power ARM microcontrollers. The former could be used as a platform for the development of interoperable gateways; the latter can be used for energy constrained applications, for instance for signal repeating or optical reading on the smart water meter side. From the communication perspective, many messaging protocols, such as Message Queue Telemetry Transport (MQTT) and Advanced Message Queuing Protocol (AMQP), have been developed as open standards and have been thoroughly designed and tested in a plethora of real life applications. They could be employed for reliable communications in smart water metering applications. Finally, a plethora of COTS open source software components, including implementations of MQTT and AMQP messaging systems, can be easily reused for IoT applications. Those components would be particularly well-suited for the realization of smart water metering systems, enabling a rapid application development approach and an impressive reduction in time to market.

## 3. Design of SWaMM

Edge Computing is a relatively recent paradigm that allows IT service developers and providers to allocate some data-processing tasks at the edge of the network on the top of different edge devices (IoT gateways, Cloudlets, Micro-Cluds, etc.), with the potential of significantly reducing services’ latencies and improve the quality of those services, and thus, enabling a better utilization of both hardware resources and network bandwidth by limiting the communications with Cloud Computing platforms. We designed SWaMM in accordance with the Edge Computing paradigm to enable data processing directly at the edge of the network, thus, exploiting the available computational resources used for the collection of the metering data and also reducing the number and the size of data transmissions over the cellular network.

According to the Edge Computing paradigm, IoT applications run on devices at the edge (IoT gateways, cloudlet, micro-cloud, etc.) to exploit the proximity of users and data and provide better QoS and QoE to the users of those applications. Exploiting the proximity of users and devices is one of the key ideas behind the realization of Edge Computing solutions. In fact, by processing partially or completely the data gathered by IoT sensors and devices at the edge is possible to create more effective and responsive services, thus, enabling a better utilization of both hardware resources and network bandwidth by limiting the communications with Cloud Computing platforms.

**Figure 1:**
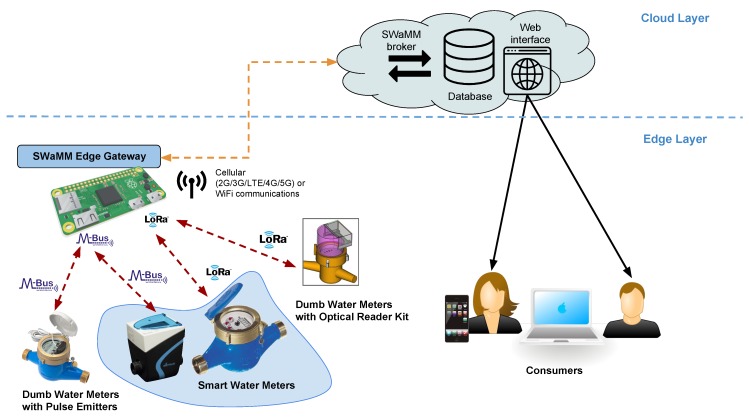
Holistic view of the SWaMM solution: from the smart and traditional water meters to the end-users (consumers and water utilities).

Figure 1 depicts the design of SWaMM and the information workflow within the middleware. At the lowest level we have the water meters (smart and dumb), which measure the consumption of the commodity (water) of the monitored accounts. As shown in Figure 1, SWaMM is capable of interfacing with different types of water meters that communicate at the edge of the network with different wireless protocols (in Figure 1 LoRa and Wireless M-Bus) and different ad hoc data formats. More specifically, Figure 1 depicts three different types of water meters: smart water meters, which were designed with radio capabilities, dumb water meters retrofitted with optical reading kits, and dumb water meters equipped with pulse emitters. Smart water meters transmit the cumulative consumption, recorded up to a certain time instant, (expressed as volume in m^3^) using different wireless protocols on configured radio frequency channels. Instead, the dumb water meters illustrated in Figure 1 represent the case of already deployed water meters not provided with automatic reading capabilities by design, but integrated with optical reader kits or pulse emitters to provide them the smart capabilities of transmitting consumption information to the SWaMM Edge Gateways located at the edge in proximity of those metering devices.

**Figure 2:**
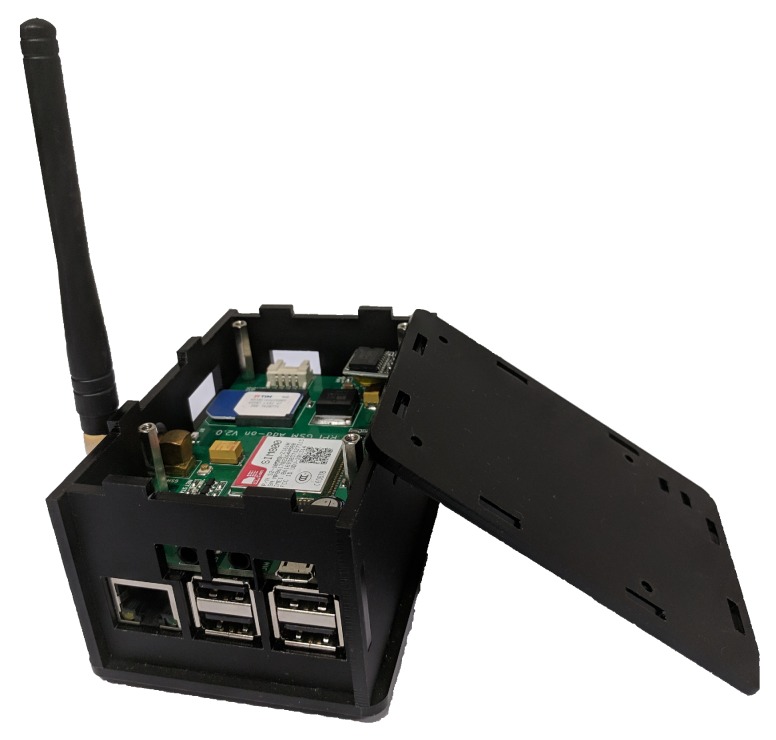
SWaMM smart metering device: SWaMM Edge Gateway, leveraging a Raspberry Pi 3 board with LTE Module.

At the other end, we have multiple SWaMM Edge Gateways running at the edge (as shown in Figure 2) that we specifically designed to receive, concentrate, and process data from the in-range available wireless meters. With regards to the smart meters currently available on the market, we developed the SWaMM Edge Gateways to receive both LoRa and Wireless M-Bus meters by carrying one or more antennae capable of receiving meters from the two different protocols. Considering the transmission power of these smart meters, the gateways are usually located in strategic points in order to maximize the number of covered smart meters and to minimize the number of required gateways.

However, we designed the SWaMM Edge Gateway without coupling its components to a specific wireless protocol, thus, enabling the easily integration of its sensing capabilities to different type of water meters and different IoT wireless protocols that would be available in the future, and thus, allowing to avoid vendor and protocol lock-in. For instance, to allow the OCR (Optical character recognition) processing at the edge, we used relatively powerful SBCs (Raspberry Pi 3) as a hardware platform for the realization of the gateways. In fact, SWaMM Edge Gateways can also analyze data from dumb water meters using OCR techniques directly at the edge to enable data collection from old meters, which do not have automated reading capabilities, thus, avoiding to transmit pictures over the cellular network to a Cloud Computing platform for elaboration.

The gateways store and pre-process the metering data at the edge before uploading via WiFi or the cellular network (3G/4G/LTE) the aggregated data to an application running on a Cloud Computing platform, which makes further elaborations of the data by means of algorithms for consumer profiling, leakages and fault detection, and so on. Data about consumption, alarms, and profiling are then made available to the end-users of the monitoring system (utility management and consumers) via a personalized web application.

With this design, we tried to achieve a comprehensive solution, which enables an automated metering data collection from different water meters available on the market and enable interoperability from a water utilty perspective that need to collect and elaborate data from its entire metering park. SWaMM offers capabilities that result in a detailed monitoring of the water distribution system capable of detecting district and/or accounts leakages, and thus, lets water utilities to optimize the water distribution system and schedule maintenance interventions.

## 4. Middleware Architecture of SWaMM

We designed SWaMM using open source technologies with the aim of creating an open solution to tackle the entire flow of a smart metering system from data collection to data elaboration. Furthermore, we exploited edge computing solutions by developing ad hoc gateways for the processing and aggregation of the metering data at the edge. In addition, having intelligent devices at the edge also enables the possibility of a granular management of the smart meters, thus, allowing to easily control all the components of the metering system.

### 4.1. SWaMM Edge Gateway Architecture

The SWaMM Edge Gateway is a data collection and elaboration kit capable of easily integrating with a wide spectrum of different types of smart meters and wireless protocols located at the edge of the network. The kit allows to collect and aggregate metering data from the nearby smart meters, to run OCR algorithms on pictures collected from old water meters, and to publish the data processed at the edge to the SWaMM elaboration platform running on the Cloud.

**Figure 3:**
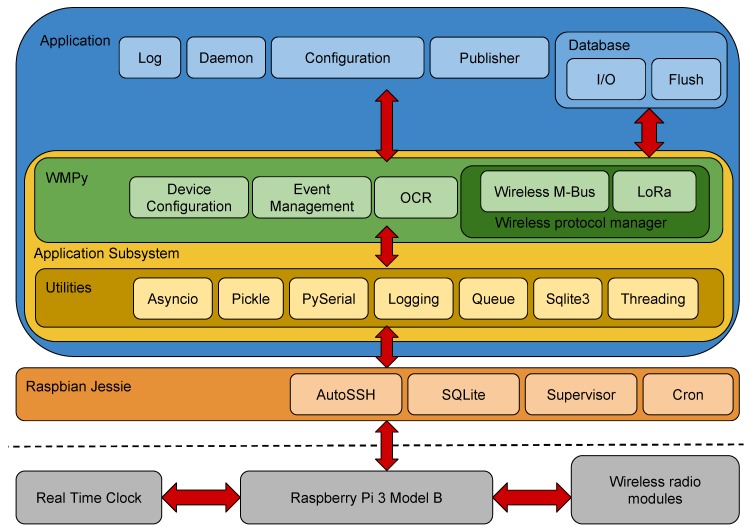
SWaMM Edge software architecture of SWaMM.

Figure 3 depicts the several tiers composing SWaMM Edge, the software architecture running on a SWaMM Edge Gateway. SWaMM Edge has been developed on several tiers to facilitate the development and the debugging of the system and at the same time to simplify future updates and/or extensions to the smart metering middleware. We chose to implement all the components using the Python programming language version 3.5.4 to exploit the wide range of available and open source libraries and the useful help from the international community.

Figure 3 provides an holistic view of SWaMM Edge’s architecture, including the blocks to deal with wireless modules, serial ports, and data-aggregation and elaboration. This platform runs on the top of Raspbian Jessie, the operating system we chose for the Raspberry Pi Model 3. Delving into the software architecture, the application subsystem is responsible to deal with the low level functionalities provided by the OS and the additional hardware, and at the other end to interface with the high-level functionalities (configuration, storage, etc.). In addition, the application subsystem provides several utilities used in the development of the smart water metering middleware. Specifically, the most important modules composing the application subsystem are:

- Logging for application monitoring and collection of the data;
- Threading and Asyncio, for the correct execution of the multithreaded application;
- Pyserial for interfacing with serial ports;
- Sqlite3 for interfacing with the SQLite relational database;
- Pickle for serializing and deserializing Python objects.

At the core of the application subsystem is the Water Management Python (WMPy) module, a library specifically designed to interface the SWaMM gateway with the wireless communication kits: Wireless M-Bus and LoRa. This library provides functionalities to deal with event management, configuration, devices monitoring, and communications management (sending and receiving messages over Wireless M-Bus and/or LoRa). The support for other meter types requires the development of a new plugin component for the Wireless protocol manager which provides the support for the specific communication protocol and data format used by that meter. In addition, another important piece composing the WMPy library is the OCR module. This module is capable of running OCR algorithms to detect the cumulative consumption of a water meter by processing the picture of its consumption wheel. To this end, we leveraged on the Google Tesseract OCR (https://opensource.google.com/projects/tesseract) software capabilities of analyzing and retrieving a text from an image, in this case the cumulative consumption value [21].

Then, on the top of the Application Subsystem we have several other software modules to implement data collection and aggregation. In detail, the more important modules are:

- Configuration management: this module keeps track of a list of authorized devices that determines whether or not a gateway has to analyze data sent by a specific smart meter. This module is also responsible to tune the configurations of the associated gateways;
- Daemons: processes running continuously and controlled by Supervisor, developed to listen for Wireless M-Bus and LoRa messages;
- Log: a logging module to keep track of the outgoing system’s events to enable history tracking and to identify bug and system faults;
- Database management: two processes for managing the database, one for saving the received data and the other one to flush the database;
- Publisher: for sending the pre-processed data (over the cellular or WiFi network) to the Cloud Computing platform.

Finally, processing the metering data is not computationally expensive since it requires only to parse and elaborate the received message. Furthermore, messages are received and buffered/stored on an in-memory buffer waiting to be processed, and thus, avoiding to lose metering data. Finally, possible limitations regarding the number of supported smart water meters mainly depend on protocol specifications, collision and interference avoidance mechanisms, and radio frequency propagation issues, e.g., the presence of obstacles that can block the propagation of the wireless transmissions [22,23].

### 4.2. SWaMM Optical Reader Kit

**Figure 4:**
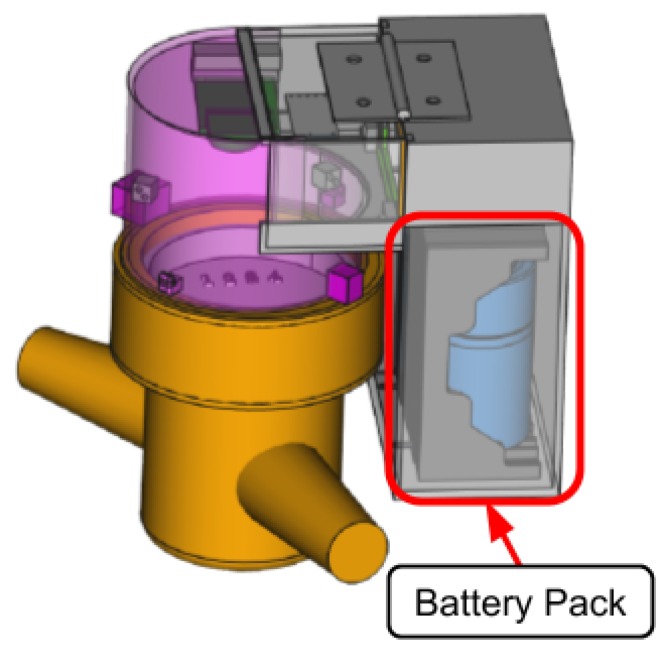
Three dimensional model of a “Dumb” water meter equipped with the SWaMM ORK module.

To enable data collection from old water meters we specifically designed the SWaMM Optical Reader Kit (ORK). The SWaMM ORK is a sort of smart cap that can be assembled on different type of dumb water meters. The SWaMM ORK provides “smartness” to old dumb meters, which do not support other smart reader mechanisms e.g., a pulse emitter, by means of a radio module that transmits the picture of the consumption wheel visible on the water meter. In detail, Figure 4 illustrates the three dimensional model of a mechanical “dumb” water meter equipped SWaMM ORK.

**Figure 5:**
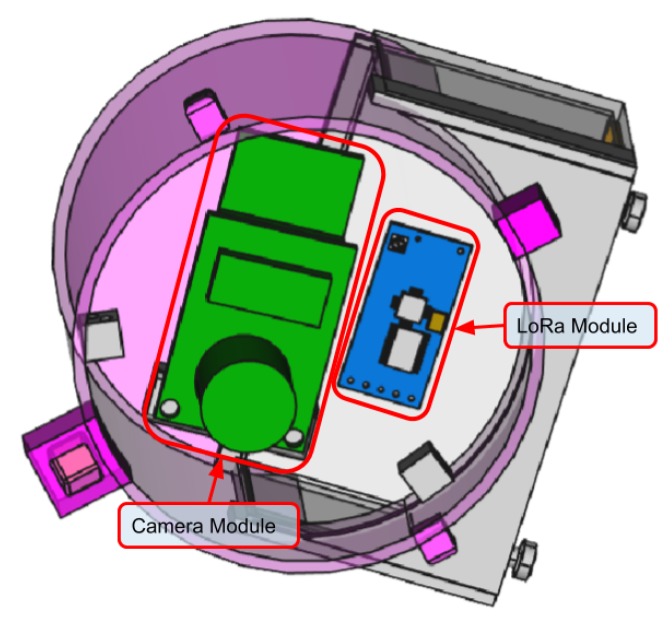
Three dimensional model of the SWaMM ORK module.

To realize SWaMM ORK we developed the illustrated prototype by analyzing the factor form of the most common used water meters to make this cap suitable for collecting data on them. As illustrated in Figure 4, this cap contains a battery powered micro-controller equipped with a camera and a LoRa radio module, for wireless transmissions. More in detail, Figure 5 depicts the three dimensional model of the cap by highlighting the camera and its factor form.

SWaMM ORK can be configured to take a snapshot of the meter’s consumption wheel with a desired frequency, e.g., each day, and to transmit the resulting JPEG image to the nearby SWaMM Edge Gateway through the LoRa radio module for elaboration. Before transmitting the picture, the SWaMM ORK converts the JPEG image in gray scale in order to reduce the size of the data to be transmitted over LoRa.

### 4.3. SWaMM Platform

**Figure 6:**
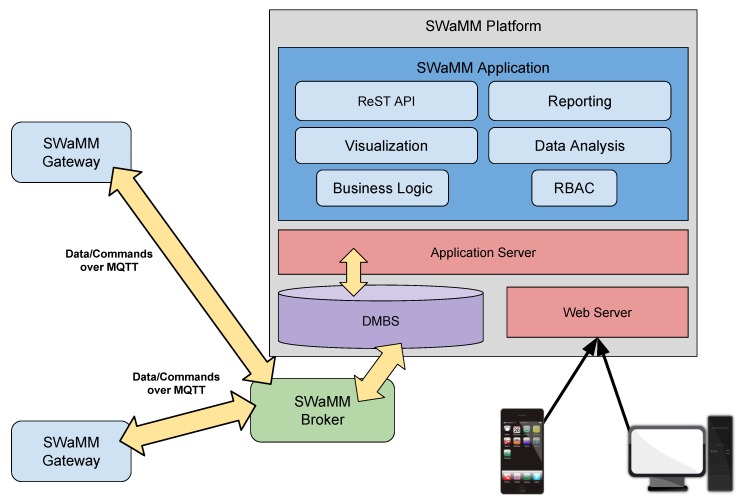
SWaMM Platform architecture.

Figure 6 illustrates the architecture of the SWaMM Platform, a comprehensive application for metering analysis and elaboration composed of seven software modules that, following the classic three-tier paradigm, uses the functions offered by a DBMS (Database Management System) for data management, and interfaces with the outside through a Web Server. The large amount of data to be stored, and subsequently analyzed, requires the adoption of tools that allow intelligent and efficient archiving, and which allow the analysis tools to quickly find the requested data. For this reason, we provided the SWaMM platform with a DBMS solution for the efficient management of data.

The Business Logic, RBAC (Role Based Access Control) and CRM (Customer Relationship Management)/ERP (Enterprise Resource Planning) integration components are the business logic layer of the SWaMM platform. The Business Logic module implements the business logic of the platform and provides the management functions of gateways, including (re)configuration and firmware upgrades.

The RBAC module implements the authorization mechanisms to access the various functions offered by the SWaMM middleware, using an approach that allows the SWaMM platform to realize different access levels, e.g., SWaMM system operators, providing a specific subset of functionality depending on the user’s role. This module is quite important considering the sensibility of water consumption information. In order to preserve the customers’ privacy only authorized subjects can have access to sensible data.

The ReST API (Representational State Transfer Application Programming Interface) component provides an access interface to the SWaMM platform functions specially designed to allow integration with other platforms and software systems. More precisely, the functions of the SWaMM platform will be accessible through the Web using an API based on the ReST architectural paradigm. To this regard, we report some example on how to interface with the SWaMM ReST API in Table 1. Using this API is possible to retrieve the information of all accounts or of a specific account, which is identified using an identification number (id). In addition, water utilities can retrieve information about water consumption, detected leakages and push software update notifications to the SWaMM Edge gateways.

This will not only automate the export of metering data collected in an appropriate format based on XML (eXtensible Markup Language), JSON (JavaScript Object Notation) or CSV (Comma-separated values), thus, facilitating integration with sophisticated and powerful Big Data analysis tools, but also accessing “programmatically” all the functions of the platform, including the management of the SWaMM Edge Gateways. The SWaMM platform also provides advanced reporting functions, able to create, both on demand and in an automated way at scheduled deadlines, detailed technical documents that effectively highlight the state of the monitored accounts.

Finally, the Data Analysis module is the module responsible for data elaboration. In particular, this module analyzes the collected and aggregated data using fault and leakage detection algorithms that we specifically designed for the management of water distribution networks. Then, users can access the elaborated results via the Web Application, which exposes a user-friendly interface to inform about consumption and the presence of leakages on the monitored accounts.

#### SWaMM Broker

The SWaMM Broker is the component of the wireless metering middleware that deals with all aspects related to the management of interactions between the gateways and the SWaMM elaboration platform. The SWaMM Broker allows a spatial and temporal decoupling of the communications between SWaMM Edge Gateways at the edge and the SWaMM platform running on the Cloud, which makes the platform significantly more robust, since the various components can continue to work even in case of temporary component failures.

In addition, we believe that the adoption of a broker-based solution has many advantages, including the ability to transmit metering data and commands over different communication protocols, such as AMQP (Advanced Message Queuing Protocol) or MQTT (Message Queuing Telemetry Transport), and the ability to enable an efficient communication between software components with remarkably different performance. Furthermore, the SWaMM Broker also takes care of saving and storing the data by interacting with the DataBase Management System. We chose to use MQTT because of its simplicity and its small overhead. In fact, a single server can support thousands of remote clients. Furthermore, MQTT allows high scalability from the point of view of the development and management of applications. We chose RabbitMQ server side because we found it reliable and fully configurable, and thus, well suited to be installed on a Cloud Computing platform. At the same time we chose Mosquitto at the client side because is open source, well documented and supported, and it is the state of the art for implementing MQTT client applications.

In addition, the SWaMM platform makes use of the broker to interact with the SWaMM Edge Gateways by sending management commands for tuning the behaviour of the smart meters associated to the gateways. The broker exchanges both data and command messages via MQTT in order to guarantee the reliability of the platform, thus, limiting errors due to components and network failures.

Finally, MQTT topics follow the style illustrated in Table 2. According to the described architecture, a SWaMM Edge Gateway both publishes and subscribes to the SWaMM broker. First, a gateway publishes the processed data regarding the cumulative consumption value of each associated account on the consumption topic according to the format described in Table 3. This message contains all the information regarding a single meter reading needed for further elaboration. We chose to use a different topic for each account since this choice simplifies data management and elaboration (data is already organized and do not need to be divided). Second, SWaMM Edge Gateways subscribe to the management topics for receiving management commands from the SWaMM platform. Here commands can be of different types such as configuration and software update. For example, if a new functionality is required, the SWaMM platform publishes a software update message to the *gateway/management/update* topic. Then, at the reception of this message, gateways will download and install the update locally.

## 5. Experimental Evaluation

Within the GST4Water project (Green Smart Technology for Water), we thoroughly evaluated our solution and proved the validity of SWaMM in the city of Gorino Ferrarese (Ferrara, Italy) [24,25]. As part of the GST4Water project, we installed the SWaMM solution over the residential accounts of the water district of Gorino Ferrarese in order to monitor water consumptions and identify possible leakages. In addition, we evaluated the performance of the OCR module implemented within the SWaMM Edge Gateway to recognize correctly the cumulative consumption values reported on old dumb water meters.

### 5.1. Field Testing with W-Mbus and LoRa Smart Meters

**Figure 7:**
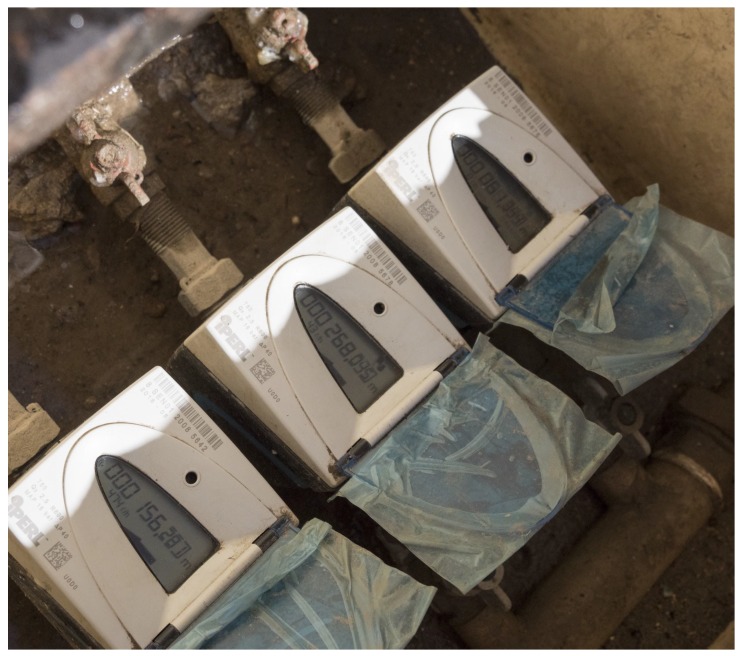
Example of an inspection pit in Gorino Ferrarese, Italy.

Part of these tests focused on the evaluation of Wireless M-Bus smart water meters to identify protocol performances, the number of required gateways, and other configurations. In detail, we used the Sensus iPerl smart water meter (https://sensus.com/products/iperl-international/), which has a built-in Wireless M-Bus module that we configured to transmit on the 868 MHz frequency a meter every 15 min. Instead, with regards to the SWaMM Edge Gateways, we configured the Raspberry Pi 3 with the IMST (iM871A-USB) USB Wireless M-Bus dongle to receive the Wireless M-Bus messages from the smart meters. In particular, we expected to experience radio frequency propagation issues due to the locations of these smart meters. In fact, as usually in Italy, these smart water meters were located in underground inspection pits as the one shown in Figure 7. At the other end, we installed (for testing purposes) the gateway inside the consumers’ houses.

The following tests report the data collected from five different locations; each one characterized with distinct peculiarities:

- Location 1: the smart meter was located inside a cast iron covered inspection pit, which was 22 m away from the gateway installed at the third floor of the house;
- Location 2: the smart meter was located inside a cast iron covered inspection pit, which was 15 m away from the gateway installed at the third floor of the house;
- Location 3: the smart meter was located inside a concrete inspection pit, which was 10 m away from the gateway installed at the ground floor of the house;
- Location 4: the smart meter was located inside a cast iron covered inspection pit, which was 20 m away from the gateway installed at the first floor of a two-family house. In particular, this location presents another issue related to presence of an house between the smart meter and the gateway;
- Location 5: the smart meter was located inside a cast iron covered inspection pit, which was 22 m away from the gateway installed at the third floor of the house;

The results reported in Table 3 regard two 36 h periods (one for Wireless M-Bus and the other one for LoRa, which correspond to 144 Wireless M-Bus packets.

As depicted in Table 3, the Wireless M-Bus protocol (configured on the 868 MHz) did not guarantee a reliable transmission of data in the above described configurations. In detail, we achieve a 65.28% delivery ratio in location 3, when the smart meter was located inside a concrete inspection pit relatively close to the gateway (10 m). On the other hand, the characteristics of location 4 resulted in a 0% delivery ratio. The other results indicate that only part of the sensed data was delivered to the SWaMM Edge Gateways (from 36.80% to 52.78%). Possible solutions to overcome these results are to increase the data collection frequency, to guarantee the collection of a sufficient amount of data, or to tune Wireless M-Bus protocol specific configurations, e.g., increase the transmission power. However, both solutions present drawbacks related to an increased battery consumption, which results in a reduced smart meters lifetime.

To solve these data delivery issues and increase the reliability of the data collection system, we used an ad-hoc designed bridge to transmit over LoRa the Wireless M-Bus messages. During these tests, we noticed a clear delivery ratio improvement, as depicted in Table 3.

According to these results, we believe that the LoRa protocol outperforms the Wireless M-Bus protocol in all those situations where the outdoor smart meters are installed in underground inspection pits or they are particularly distant from the gateways where the presence of natural shielding conditions considerably reduce the range of those smart meters.

### 5.2. Water Leakage Detection

We collected and analyzed the consumers’ consumption data to demonstrate the validity of SWaMM as smart water metering solution. Using the collected data, the SWaMM elaboration platform running on the Cloud can identify leaks of different sizes and behavior at household level: from large leakages due to broken pipes, to small leakages due to the faulty operation of sanitary appliances. Furthermore, the collected data can be used to characterize the leak level in the distribution network. Indeed, leaks, both at pipe network and household level, nowadays represent an important problem and huge effort is required to characterize and reduce these leakages through development and application of new approaches [26,27,28,29]. In particular, in the case study considered at household level a total of about 200 leakages were identified, of which the 25% corresponding to large water leakages of more than 10 L/h and the 75% corresponding to small water leakages in the range from 1 to 10 L/h.

**Figure 8:**
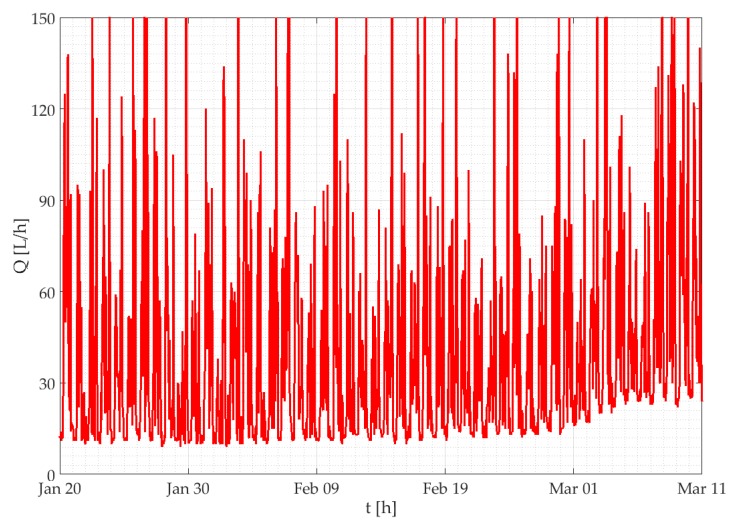
Hourly incremental consumption trends of a user affected by a large leak.

**Figure 9:**
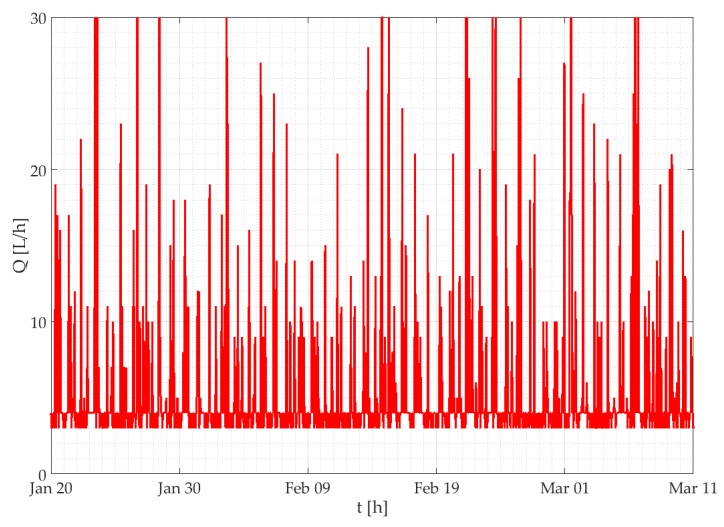
Hourly incremental consumption trends of a user affected by a small leaks.

The SWaMM elaboration platform detects water leakages through an empirical algorithm based on the analysis of the hourly flow rates time series. This criterion has been defined taking into consideration the typical water consumption pattern of residential accounts. With this regard, a residential account is characterized by periods of higher consumption (peaks), which mainly occurs during the day, alternating with periods when water consumption decreases to zero (mostly during the night). Instead, if an account is characterized by a leak, the smart water meter will detect a continuous consumption, which will be marked as leakage by the monitoring system (incremental consumption that never stops during the whole day). Thus, the algorithm is set up to assess the presence/absence of leakages inside residential houses by looking for non-consumption in certain periods of the day. Examples of detected leakages are the ones depicted in Figure 8 and Figure 9, which show a large water leakage due to a damage of the hydraulic system and a small water leakage due to a failure of a toilet box, respectively. In both Figures the water leakage is identified by the incremental consumption that never goes to zero.

**Figure 10:**
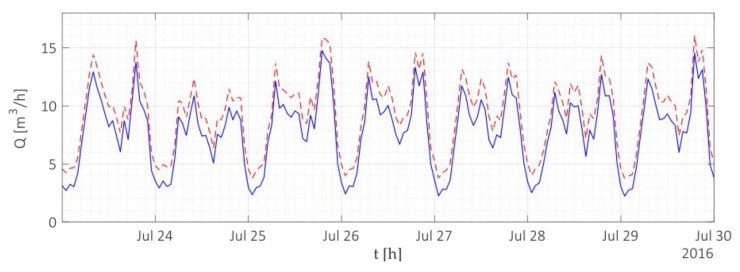
Water balance with reference to the district metered area of Gorino Ferrarese (FE) in July 2016.

On the other hand, water utility managers have the opportunity to analyze the collected data to optimize the management of the water supply network and to reduce real water loss levels, by applying the water balance method using the high resolution data. In fact, managers can estimate the leak level of the distribution network by monitoring the incoming and outgoing discharges from well-defined portions of the distribution network (using Supervisory Control And Data Acquisition (SCADA) systems) and by combining this information with the consumption trend of all users belonging to the same area. For example, Figure 10 shows the water balance with reference to the water distribution network of Gorino Ferrarese (FE). In detail, the blue line indicates the consumption of all users belonging to the monitored area, while the red line (dashed line) indicates the result of the combination of the incoming and outgoing discharges from the same area, measured through the proprietary SCADA system. If the distribution network does not present leakages, the two lines (illustrated in red and blue) should overlap. However, as shown in Figure 10, the distribution network is affected by water leak, since the combination of incoming and outgoing discharges at the district is always greater than the monitored consumption of all users during the illustrated period, and thus, identifying a water leakage in the water distribution network.

**Figure 11:**
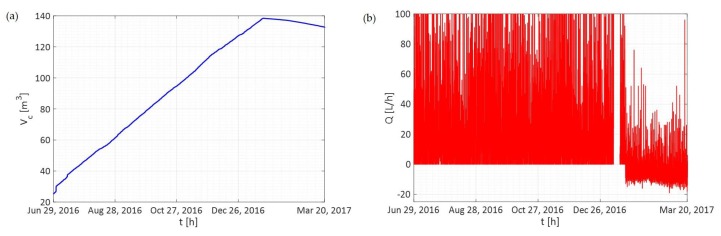
Totalized consumption trend measured by a faulty smart meter (**a**) and incremental consumption trend reported by the Cloud platform (**b**).

In addition, the water utility has the opportunity to identify faulty smart meter devices immediately, reducing the apparent water losses due to measurement errors. For example, Figure 11a illustrates the total consumption trend of a residential account starting to decrease, and thus, indicating faulty data (the total consumption cannot decrease over time). This result is also confirmed in Figure 11b, where the incremental consumption values are negative, which is impossible since the discharge cannot assume negative values.

### 5.3. Interfacing with Dumb Water Meters

**Figure 12:**
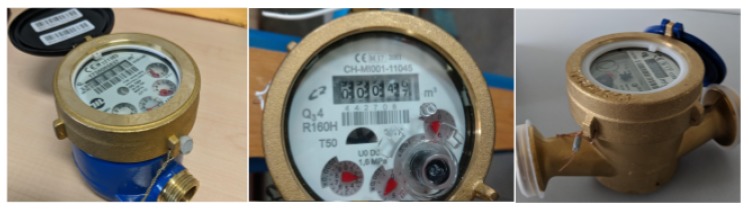
Three types of mechanical dumb water meters.

We conducted several tests to evaluate the performance and the quality of the OCR module in detecting the cumulative consumption on pictures taken from different types of water meters. Considering the wide variety of water meters deployed on the field, we consider for the scope of these tests only three types of water meters, which are the most common used in the city of Ferrara, Italy. Figure 12 depicts three different types of mechanical water meters and how each water meter is characterized with a particular form-factor and appearance, and thus, tending to influence the text recognition results of the OCR processing.

**Figure 13:**
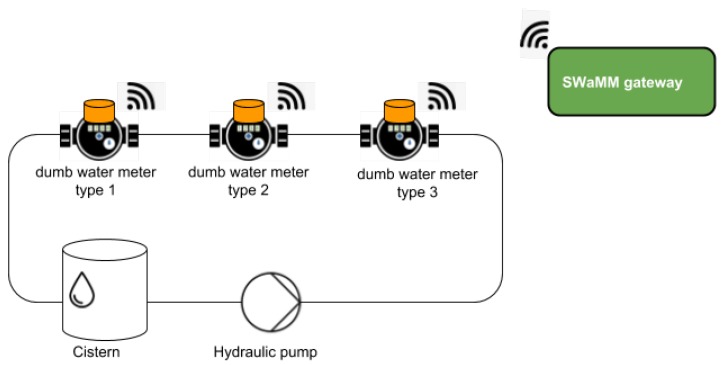
The hydraulic network setup.

**Figure 14:**
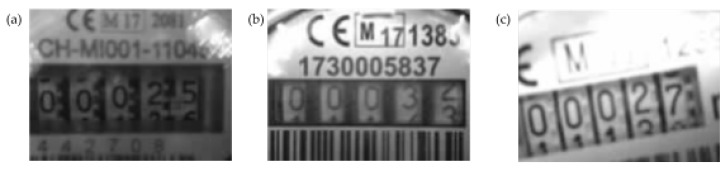
Different radials example. Dumb Meter 1 (**a**) Dumb Meter 2 (**b**) Dumb Meter 3 (**c**).

During these tests, we evaluated SWaMM ORK and the efficiency of the OCR module on dumb water meters put into operation, to verify its functionality during the time. To this end, we realized a simplified hydraulic network, as the one illustrated in Figure 13. Into this hydraulic network, an hydraulic pump puts the water meters at work, meanwhile the ’reader’ components works continuously taking snapshots and transmitting pictures to the SWaMM Edge Gateway, located nearby the hydraulic network. Figure 14 depicts an example of images collected using the SWaMM ORK on the gateway side. As illustrated in Figure 14, SWaMM ORK converts light compressed JPEG into grayscale pictures before the transmission, in order to reduce the size of the data to be transmitted over LoRa and transmission time consequently.

On the SWaMM Edge Gateway side, the OCR module is initially instructed on the position of the digits to process each one individually. Initially, we used the standard Tesseract engine of Google Tesseract 4.0. Unfortunately, applying the standard Tesseract engine on the meter’s digits did not produce extremely brilliant results. A first cause was due mainly to the fact that Tesseract’s neural network is trained to identify all the possible characters commonly used for writing (alphanumeric) for a multitude of fonts. In our case, instead, the interest have to be focused only for the specific fonts used on the water meters’ consumption wheels. Another fundamental motivation is depicted in Figure 14b. The consumption wheel does not always show “entire” digits, but sometimes it could show a “digit transaction” state, which is a digit between two consecutive values. For example, in the least significant digit of Figure 14b, we find a transaction between value 2 and 3, meanwhile the remainder digits are all “entire” numbers.

For this reason, we decided to re-train the Tesseract LSTM neural network, using the pictures collected from the dumb water meters as a training dataset. After two days of system operation, we collected about 1400 images for each dumb meter, which included both entire digits and “digit transactions”. Using the re-trained neural network, we achieved an accuracy of the results between 80 and 90 percent.

However, we tried to further improve the text recognition quality results. To this end, we designed a specific software component that works following the Tesseract processing, implementing an algorithm we called “validation process”, which tries to improve the precision of the results obtained using Tesseract. We developed this validation algorithm according to the suggestions of the water utilities we collaborated with during the development and field testing of SWaMM. This algorithm tries to improve the text recognition accuracy by limiting the identification of result not corresponding to the reality, using the know-how of the typical average water consumption of residential accounts. In this way, the algorithm can validate or possibly correct the Tesseract results. For instance, in the event that a proposed result is not compatible with domestic consumption patterns, the validation algorithm runs a series of checks to identify what caused the possible recognition error. Errors can be differentiated in single digit recognition error (marginal) or multi-digit recognition error (substantial). In the first case, the algorithm tries to correct the possible error by leveraging on the Tesseract capabilities, while in case of substantial errors, the algorithm adopts a more conservative approach and keeps the value of the previous correct reading since it is not possible to interpret the result of Tesseract processing.

To demonstrate the functionality and utility of the validation process algorithm, we conducted a series of tests for the dumb meter of type 1, each time selecting 40 different images that depicted the consumption trends of this meter. In Table 4, we present the obtained results. The first case reports the recognition results obtained using only the re-trained Tesseract network. Instead, the second case shows the results applying our validation algorithm on the re-trained Tesseract outputs. Finally, the last two columns represent indicators that we calculated to estimate the size of the algorithms errors. In detail, deltaT represents the sum of the differences, expressed in m^3^, between the Tesseract results and the correct ones. At the same way, deltaP represents the sum of the differences, expressed in m^3^, between the validation process results and the correct ones.

As shown in Table 4, the validation process has a final 88.63% medium percentage of correct results of all the tests performed. Although it slightly improves the percentages of the medium re-trained Tesseract precision (87.66%), we find a good improvement on the errors size, from an average error of 2392 m^3^ using only the re-trained Tesseract, to only 5 m^3^ of average error for the validation process. These results are acceptable for water bill calculation. In fact, considering that billing procedures are usually performed monthly, or sometimes even bimonthly, the amount of readings pertaining to the billing time window tend to even out the error in a single cumulative water consumption reading. Therefore, an average error of 5 m^3^ for a single cumulative water consumption reading is relatively small and well within the acceptance window.

Finally, we calculated using the *process_time* function (process_time function, https://docs.python.org/3/library/time.html#time.process_time) of the Python time module that the entire processing of the consumption wheel’s picture (image elaboration and OCR processing) takes about 1261 ms (milliseconds) on the Raspberry Pi 3. This means that a SWaMM Edge Gateway can process around 7100 readings from Optical Reading Kits per day. Considering one or two readings per meter per day (sensible reading frequencies, which represent a good tradeoff between energy consumption at the Optical Reading Kit level and “observability” of the water consumption process) this means that a gateway could respectively support 7100 and 3550 water meters. However, radio frequency propagation issues and bandwidth limitations impact on the amount of data that can be transmitted and consequently reduce the number of water meters that can be served by a single gateway. These results seem to confirm the validity of the retrofitting solution in those situations in which replacing a traditional water meter with a new one is difficult. Therefore, the OCR processing does not present a limiting factor on the SWaMM Edge Gateway.

## 6. Discussion

In the near future, the growing need for environmental sustainability is expected to push the need for smart metering solutions that avoid the waste of natural resources and increase the population awareness. To this end, smart water metering solutions provide many advantages for both consumers and water utilities. On the one hand, consumers can look over the time series of totalized and incremental consumption and the volume of water required during the last 24 h, thus, allowing real-time monitoring of their consumption and consequently to adopt more environmental friendly (virtuous) behaviors, thus, avoiding bad habits and waste of water. In addition, consumers can benefit of a more transparent water bill and avoid possible inconveniences caused by refund procedures, since the consumption is always determined on the basis of real data instead of a general forecast. Furthermore, this solution offers consumers the opportunity to receive an alarm message when the smart water metering platform detects leaks within the consumer’s property, and thus, reducing waste of water even more.

Another advantage of using smart water metering platform is that the collected data can be used to implement water demand forecasting models for the entire district monitored areas. As a result, the general utility would be able to optimise the distribution network management by controlling the pressures of the distribution network and ensuring a more efficient management of the entire water district (control valves and pumping systems). In addition, a smart monitoring system also allows to reduce energy consumption and water losses, which are usually connected to an excessive amount of pressure in the distribution network. Finally, water utilities can make use of the collected data to plan marketing strategies, provide feedbacks on their water consumption or recommendations on personalized water-saving practices.

Our extensive field testing experience with SWaMM showed that the design of an efficient and automated smart water metering solution requires an intelligent analysis of the performance of wireless protocols, the optimal placement of gateways, and a thorough testing and evaluation phase. As we reported in the previous sections, some wireless protocols may perform significantly better than others when physical limitations can interfere with data collection procedures. On the other hand, protocols that enable longer ranges usually require a bigger power consumption, and thus, limiting the average lifetime of smart meters.

In the smart water metering market, interoperable wireless middleware solutions such as SWaMM, based on open technologies, both at the software and the hardware levels, and capable of dealing with different communication protocols and data formats, represent a key enabling technology. In fact, unlike stovepiped closed proprietary systems, SWaMM does not force to use a specific type of water meter, neither at the radio communication protocol nor at the meter type (smart or dumb) level, and allows water utilities to choose among different water metering technologies and to switch to different vendors. We believe that this capability will gain importance over the next years when new manufacturers will enter into the smart metering market, thus, influencing the heterogeneity in metering parks composition.

Finally, the use of SWaMM might open the market to 3rd party companies that operate between consumer and water utilities. These companies can operate as intermediary and offer metering solutions for collecting and elaborating metering consumption data, and thus, opening scenarios where the metering infrastructure is sold as a service.

## 7. Related Work

Smart metering is an active topic in research literature. Many efforts have been done to propose open and interoperable solutions for the smart metering of water and other commodities. This work [30] analyzes the applicability of the LoRa protocol for data collection purposes in a smart electricity metering system.

With focus on wireless protocols for smart metering solutions, [31] provides an accurate study of LP-WAN protocols for IoT, by describing performances of (LoRaWAN, NB-IoT, etc.) in different conditions such as small and large cities, rural areas, and so on.

In [32], Simmhan et al describe a service-oriented and data-driven architecture for Smart Utilities. In particular, this work describes, proposes, and evaluates protocols and solutions for IoT infrastructures in Smart Cities, also presenting real applications developed within a campus in Bangalore, India. Another interesting work is the one described in [33], in which the authors propose a smart metering infrastructure for water, electricity, and gas, by discussing possible wireless protocols for data collection, clustering techniques and prediction models for data elaboration and forecasting.

In [34], the author presents an interesting survey on smart grid and smart grid communication. The survey discusses smart grid and related technologies also by giving an exhaustive example of a smart energy infrastructure. Furthermore, the author analyzes wireline and wireless smart metering communication technologies needed to implement a measurement system, and the possible security threats and issues related to a smart grid infrastructure.

A smart water metering architecture is discussed in [35]. In this work, the authors describe an IoT architecture for smart water management by proposing a solution called the MEGA model. This work focuses mainly on the architecture and the interactions within the subsystem, but it also presents an implementation scenario in which the proposed solution is evaluated. [36] presents instead an IoT smart water monitoring system based on COTS hardware (Arduino and Raspberry Pi) to monitor the water level of tanks located across a campus.

SWaMM differs from the other solutions by proposing a comprehensive wireless middleware for smart water metering, which tackles the entire information flow of the metering data, from the sensing to the elaboration. Furthermore, we designed SWaMM to be open and interoperable with a wide range of wireless protocols, thus, enabling the integration of more reliable and innovative solutions.

## 8. Conclusions

We presented a comprehensive smart water metering solution that aims at tackling the heterogeneity of protocols and standards in a competitive and proprietary market and to avoid vendor and protocol lock-in. The core of this solution is SWaMM, a wireless IoT middleware for the collection and elaboration of metering data from a wide range of smart water meters. In addition, purposely developed Optical Reading Kits allow to integrate SWaMM with dumb water meters. SWaMM was thorougly tests on the field, also in the context of a full scale deployment in Gorino Ferrarese, Italy as part of the GST4Water project. The results obtained demonstrate the effectiveness of our middleware in reducing the waste of commodities over the water distribution network, by means of an accurate tracking and monitoring of consumptions.

Finally, let us note that, while SWaMM was specifically designed for smart water metering applications, its flexible design allows the middleware to be easily adopted for the smart metering of other commodities. In fact, SWaMM Edge Gateways could be used to collect data from other smart meters such as electricity and gas, as far as these smart meters will use supported wireless protocols. We believe that other IoT low power wireless protocol such as NB-IoT should be investigated and proved on the field. With this regard, we specifically designed SWaMM to be open and interoperable to future protocol extensions.

In addition to the experimentation with new water meter models and more sophisticated water leakage detection algorithms, future works will include the development of a dedicated OCR solution for dumb water meters that could provide better accuracy with respect to Tesseract, and the realization of a significantly improved version of the Optical Reader Kit, based on a low-power computing platform that allows it to operate for around 2 years on a couple of AA batteries.

## Figures and Tables

**Table 1:** SWaMM ReST API.

URI	HTTP Action	Behaviour
accounts/	GET	retrieve the account collection
accounts/{data}	POST	insert a new account
accounts/	PUT	replace (or create) the account collection
accounts/	DELETE	delete the account collection
accounts/id	GET	retrieve the account with the id
account/id	PUT	replace the account with id
accounts/id	DELETE	delete the account with id
consumption/accounts/id	GET	retrieve the account’s consumption information
leakages/accounts	GET	retrieve information about all detected leakages
leakage/accounts/id	GET	retrieve information about leakages on account with id
management/gateways/status	GET	retrieve gateways status information
management/gateways/status/id{message}	GET	retrieve status information of gateway with id
management/gateways/update{message}	POST	push an update notification to gateways

**Table 2:** Topic structure for MQTT messages.

Information	MQTT Topic
cumulative consumption value related to account number	consumption/account_number
update commands	gateway/management/update
configuration commands	gateway/management/configuration

**Table 3:** Wireless M-Bus and LoRa data delivery results.

Location	Wireless M-Bus Delivered Packets (%)	LoRa Delivered Packets (%)
Location 1	53 packets—36.80 %	133 packets—92.36 %
Location 2	58 packets—40.28 %	137 packets—95.14 %
Location 3	94 packets—65.28 %	141 packets—97.92 %
Location 4	0 packets—0 %	121 packets—84.02 %
Location 5	76 packets—52.78 %	135 packets—93.75 %

**Table 4:** Comparision between re-trained Tesseract network and validation process for dumb meter of type 1.

Re-Trained Tesseract		Validation Process			
**Errors**	**Right Results**	**Errors**	**Right Results**	**DeltaT (m^3^)**	**DeltaP (m^3^)**
15.79%	84.21%	10.53%	89.47%	469	4
6.9%	93.1%	3.45%	96.55%	69	1
17.65%	82.35%	8.82%	91.18%	10,022	5
6.06%	93.94%	6.06%	93.94%	600	2
10.00%	90.00%	12.5%	87.5%	605	6
18.75%	81.25%	15.63%	84.38%	622	6
8.57%	91.43%	5.71%	94.29%	904	3
12.50%	87.50%	18.75%	81.25%	605	8
15.79%	84.21%	23.68%	76.32%	17	12
11.43%	88.57%	8.57%	91.43%	10,006	4
**12.34%**	**87.66%**	**11.37%**	**88.63%**	**2392**	**5**

## Abbreviations

The following abbreviations are used in this manuscript:

IoT	Internet-of-Things
CADF	Consorzio Acque Delta Ferrarese
LoRa	Long Range
OCR	Optical character recognition
SWaMM	Smart Water Metering Middleware
DBMS	database management system
RBAC	Role Based Access Control
MQTT	Message Queuing Telemetry Transport
LoraWAN	Low Power WAN Protocol for Internet of Things
AMQP	Advanced Message Queuing Protocol
AMR	Automatic Meter Reading
RMR	Remote Meter Reading
CAPEX	Capital expenditure
OPEX	Operating expense
NB-IoT	Narrowband IoT
COTS	Commercial Off The Shelf
SBC	Single-board Computer
WMPy	Water Management Python
ReST	Representational State Transfer
API	Application Programming Interface
SCADA	Supervisory Control And Data Acquisition
